# The rectal mucosal immune environment and HIV susceptibility among young men who have sex with men

**DOI:** 10.3389/fimmu.2022.972170

**Published:** 2022-10-20

**Authors:** Cassie G. Ackerley, S. Abigail Smith, Phillip M. Murray, Praveen K. Amancha, Robert A. Arthur, Zhengyi Zhu, Ann Chahroudi, Rama R. Amara, Yi-Juan Hu, Colleen F. Kelley

**Affiliations:** ^1^ The Hope Clinic of the Emory Vaccine Research Center, Division of Infectious Diseases, Department of Medicine, Emory University School of Medicine, Decatur, GA, United States; ^2^ Department of Pediatrics, Emory University School of Medicine, Atlanta, GA, United States; ^3^ Emory Integrated Computational Core, Emory University, Atlanta, GA, United States; ^4^ Department of Biostatistics and Bioinformatics, Rollins School of Public Health, Emory University, Atlanta, GA, United States; ^5^ Emory National Primate Research Center, Emory University, Atlanta, GA, United States; ^6^ Center for Childhood Infections and Vaccines of Children’s Healthcare of Atlanta, Emory University, Atlanta, GA, United States; ^7^ Department of Microbiology and Immunology, Emory University, Atlanta, GA, United States

**Keywords:** HIV transmission, YMSM, rectal mucosa, mucosal immunity, microbiome, viral replication

## Abstract

Young men who have sex with men (YMSM) represent a particularly high-risk group for HIV acquisition in the US, despite similarly reported rates of sexual activity as older, adult MSM (AMSM). Increased rates of HIV infection among YMSM compared to AMSM could be partially attributable to differences within the rectal mucosal (RM) immune environment associated with earlier sexual debut and less lifetime exposure to receptive anal intercourse. Using an *ex vivo* explant HIV challenge model, we found that rectal tissues from YMSM supported higher levels of p24 at peak viral replication timepoints compared to AMSM. Among YMSM, the RM was characterized by increased CD4+ T cell proliferation, as well as lower frequencies of tissue resident CD8+ T cells and pro-inflammatory cytokine producing CD4+ and CD8+ T cells. In addition, the microbiome composition of YMSM was enriched for anaerobic taxa that have previously been associated with HIV acquisition risk, including *Prevotella, Peptostreptococcus*, and *Peptoniphilus*. These distinct immunologic and microbiome characteristics were found to be associated with higher HIV replication following *ex vivo* challenge of rectal explants, suggesting the RM microenvironment of YMSM may be uniquely conducive to HIV infection.

## Introduction

HIV transmission rates for young men who sex with men (YMSM), ages 13 to 24 years, are disproportionately high, accounting for 25% of new HIV cases among all MSM in 2018 (1). While some individual risk behaviors, including earlier sexual debut, partner concurrency, and selection of older partners, have been associated with increased HIV transmission risk among YMSM (2–4), there may also be biological determinants contributing to this disparity (5). An analysis of per contact risk of HIV seroconversion among MSM with HIV seropositive partners demonstrated a significantly higher per contact rate for YMSM (< 25 years) engaging in unprotected receptive anal intercourse (RAI) compared to older MSM (> 30 years) despite a lower mean number of reported sexual contacts (7.1 vs 10.3, respectively) (5). These findings suggest that there may be host mucosal factors among YMSM, such as a distinct rectal mucosal (RM) immune environment and gut microbiome composition, that facilitate HIV transmission.

Significant variability in the microenvironments of the rectum and the female genital tract likely accounts for the 18-fold difference in HIV transmission risk at these sites (6–8), including the gut cellular composition that contains more than 60% of all T cells in the body, many of which are primary target cells for HIV (9, 10). Our group has previously shown that condomless RAI can influence the RM adaptive immune compartment resulting in higher frequencies of Th17 cells, greater CD8+ T-cell proliferation, and increased pro-inflammatory cytokine production among MSM engaging in RAI compared to Controls with no history of anal intercourse (11). A distinct microbiota enriched for *Prevotellaceae* has also been described among MSM by our group and others (11–13). Yet, it remains unclear whether this unique rectal immune environment is also representative of YMSM, who may have more recent sexual debut and less lifetime exposure to RAI compared to older males.

Aging is associated with numerous immunologic changes, including decreased frequencies of naïve T cells in blood and lymphoid tissues, resulting in a more restricted, terminally differentiated T cell repertoire (14, 15), as well as a reduction in T cell cytokine production and B cell activity (16). From a clinical perspective, timing of pathogen transmission can significantly alter the immune system’s targeted response, as demonstrated by an increased risk for development of chronic hepatitis with exposure to hepatitis B virus earlier in life and a greater potential to manifest multisystem inflammatory syndrome in children (MIS-C) following infection with SARS-CoV-2 (17, 18). It is not known whether the immune microenvironment characteristic of younger people could influence mucosal HIV susceptibility. The aim of this study was to characterize differences in the immune phenotype and function of RM CD4+ and CD8+ T cells and in the microbiome between HIV-negative YMSM, adult MSM (AMSM), and Control males who had never engaged in RAI and to assess associations with HIV viral replication using an *ex vivo* rectal explant challenge model.

## Materials and methods

### The clinical cohort

Healthy, HIV-negative, STI-negative MSM and Controls were recruited from Atlanta, Georgia from August 2017 through January 2019. Eligible participants included YMSM (18-21 years, n=32) following receptive anal sexual debut, AMSM (≥35 years, n=33) who engage regularly in RAI, and Control males (≥35 years, n=12) who had never engaged in anal intercourse. The inclusion criteria for YMSM required RAI with at least one lifetime partner, which aligns with data suggesting that anal sexual debut most often occurs during late adolescence, typically between 16 and 18 years of age (19, 20). In contrast to YMSM, AMSM reported engagement in frequent RAI, defined as at least 5 years with ≥ 12 episodes of RAI per year, to select for a regular pattern of sexual behavior that would support the study of a distinct RM immune environment compared to YMSM with more recent sexual debut. Control males reported no prior history of RAI. The exclusion criteria included men currently on HIV pre-exposure prophylaxis (PrEP), a history of inflammatory bowel disease or other condition of the gastrointestinal tract, a history of uncontrolled bleeding diathesis, a current diagnosis of a rectal STI, and use of immunosuppressive agents. All study participants presented for a screening visit that included informed consent, a brief medical history, physical examination, rapid HIV testing, and rectal STI testing for gonorrhea and chlamydia (GC/CT). A brief sexual history questionnaire was completed by all participants engaging in RAI. Participants returned for a subsequent visit involving the collection of rectal pinch biopsies between 3 to 10 cm from the anal verge *via* rigid sigmoidoscopy without sedation or prior bowel preparation. Eligible participants were linked to PrEP care upon biopsy completion. The Institutional Review Board at Emory University approved this study.

### Rectal mucosal mononuclear cell (MMC) phenotyping

Rectal mucosal pinch biopsies were processed by collagenase IV digestion to separate mucosal mononuclear cells, as has been previously described (21). Isolated cells were then stained with LIVE/DEAD marker (Invitrogen L34957) and antibodies to CD45 (Tonbo Biosciences, 25-0459-T100), CD3 (BD, 563800), CD4 (BD, 563737, RRID : AB_2687486), CD8 (BioLegend, 344724, RRID : AB_2562790), CD45RA (BD, 560675, RRID : AB_1727498), CCR7 (R&D, FAB197F; RRID : AB_2259847), CD69 (BioLegend, 310938, RRID : AB_2562307), CD103 (BioLegend, 350222, RRID : AB_2629651), CCR5 (BioLegend, 359112, RRID : AB_2562964), α4β7 (NHP reagent resource, A4B7-APC), Ki67 (BioLegend, 350506, RRID : AB_2563860), FOXP3 (BioLegend 320126, RRID : AB_2564025), and CD25 (BioLegend 356104, RRID : AB_2561861). Cell populations were acquired on a LSR-Fortessa and analyzed with FlowJo 10.5.3 (Treestar Inc., CA). MMCs were first identified with expression of CD45+ and CD3+ and then subdivided into CD4+ and CD8+ subsets. Naïve and memory subsets were identified based on expression of CCR7 and CD45RA. CD69 and CD103 markers were used to define T_RM_ and non-T_RM_ subsets. Subsequently, these cell subsets were examined for phenotypic markers of proliferation (Ki67) and HIV susceptibility (CCR5, α4β7). All subsets were gated with either total CD4+ or CD8+ T cells as the parent population. If there were fewer than 800 events acquired, the subsequent subpopulation data from that specimen was excluded from all further analyses.

### Intracellular cytokine staining

A minimum of 500,000 rectal MMCs were stimulated with 25 ng/ml of PMA and 500 ng/mL of Ionomycin in the presence of Brefeldin A (5 μg/ml; Sigma-Aldrich) and Golgi stop (0.5 μl/ml; BD Pharmingen). Incubation and surface staining were performed as previously prescribed (11). Cells were stained for intracellular cytokines with IL-17A (Invitrogen, 12-7179-42, RRID: AB_1724136), IFN-γ (BioLegend, 502522, RRID: AB_893525), and TNF-α (BioLegend, 502948, RRID: AB_2565858) antibodies. Cytokine secretion from unstimulated and stimulated cells was assessed using flow cytometry. Subsets were gated with total CD4+ or CD8+ as the parent population. If fewer than 500 events were acquired from the stimulated specimens, the specimen was classified as a non-response to stimulation and excluded from all further analyses.

### *Ex vivo* explant challenge experiments

Three fresh rectal biopsy specimens from each participant were exposed to HIV-1 BaL (10^2.8^ TCID_50_ in a volume of 250 µL media) for 2 hours, washed extensively, and placed on collagen rafts for the 18-day incubation period, as described in detail previously (22). Culture supernatant was collected from each well at days 3, 7, 10, 14, and 18 for p24 concentration analysis, and the well was replenished with 700 μl of fresh complete media. The p24 concentrations were quantified by ELISA (ABL, Inc., 5447) according to the manufacturer’s instructions, and the values were normalized to biopsy weight.

### Herpes virus ELISAs

Commercial ELISA kits were used to quantify serum cytomegalovirus (CMV) IgG, Herpes simplex virus type 1 (HSV-1) IgG and Herpes simplex virus type 2 (HSV-2) IgG antibodies (Gold Standard Diagnostics, Davis, CA) according to the procedure described by the manufacturer.

### Microbiota sequencing

Rectal mucosal swabs (BD, 220145) were collected through the sigmoidoscope at ~8-10 cm from the anal verge. Using primers selected to optimize overall coverage and phylum spectrum (23), DNA was extracted and then amplified targeting the V3/V4 regions of the 16S gene according to the standard, validated 16S Metagenomic Sequencing Library Preparation workflow for the Illumina MiSeq System (https://support.illumina.com/documents/documentation/chemistry_documentation/16s/16s-metagenomic-library-prep-guide-15044223-b.pdf). Final 16S libraries were ~464 base pairs (bp) in length and were sequenced on an Illumina MiSeq using MiSeqv3 600 cycle chemistry (Illumina; MS-102-3003) at a loading density of 6-8 pM with 20% PhiX, generating ~20 million, 300 bp paired-end reads. Standard reagents were used throughout DNA extraction with positive (*Escherichia coli*) and negative (water) controls. Furthermore, additional positive mock community controls and negative controls were used in the PCR amplification process. DNA was sequenced according to methods described previously (24). Raw amplicon sequence reads were evaluated for quality control (QC) using the Fast QC suite with MultiQC (25, 26) and were then processed using Quantitative Insights into Microbial Ecology (QIIME2 v2021.2) (27). The Divisive Amplicon Denoising Algorithm 2 (DADA2) package (28) was used within QIIME2 to denoise and dereplicate all paired-end sequences and to create the feature table of amplicon sequence variants used within QIIME2. DADA2 parameters were chosen to trim the first 30 bp and to truncate both paired-end reads at position 240. Taxonomic assignment was performed *via* QIIME2 and the data were aligned to GreenGenes (v13_8) (29) and Silva (v132) (30) using the qiime taxonomy modules.

### Statistical analyses

Demographic and sexual behavior characteristics were compared between study groups using the Wilcoxon rank-sum test for continuous variables and the chi-squared or Fisher’s exact test for categorical variables, as appropriate. To examine differences in immune cellular subsets by study group, we first utilized a discovery approach using ordinated samples in the principal components analysis (PCA) plot, which allows visualization of clustering by study groups and utilized linear decomposition modeling (LDM) (31) to produce a *P* value for assessing the group differences of the overall cellular subsets. We also used LDM to produce individual *P* values for assessing differences of individual subsets between groups, and adjusted *P* values that account for multiple comparisons while controlling for false-discovery-rate (FDR) at nominal level 20%. The LDM essentially fits a linear model for the cellular subset data as the outcome and regresses it on continuous traits or categorical group variables while adjusting for potential confounders; however, it differs from standard linear regression in that it uses permutation-based *P* values to account for non-normally distributed cellular subset data. For these analyses, data for each cell subset were normalized to have mean zero and standard deviance of one; missing data were imputed by the mean of the observed data in the same cellular subset (refer to **Supplemental Materials** for additional details). Next, we examined the top 10 rectal cellular subsets identified by the LDM method, and the subset frequencies were compared between study groups using the Kruskal-Wallis one-way analysis of variance (ANOVA) test with Dunn’s multiple comparisons test assessing for differences in sum of rank between groups with correction for multiple pairwise comparisons.

The median log area under the curve (logAUC) p24 values and the p24 concentrations, normalized by biopsy weight, from each day of assessment were compared between study groups by the Kruskal-Wallis one-way ANOVA test followed by Dunn’s multiple comparisons test. For visualization of the overall difference in RM cellular subsets based on p24 concentrations, a PCA plot was constructed, with samples dichotomized above or below the population median p24 logAUC. We then examined associations between the top 10 cellular subsets identified by LDM as being different between groups and p24 concentrations by displaying the age-adjusted cellular subset values (by fitting a linear regression of cellular subset values on age and obtaining the residual and log-transforming the cellular subset values if they are left-skewed) and the age-adjusted p24 values in a scatter plot and calculating the Spearman’s rank-order correlations and the corresponding p-values. As there were some differences in cell subset frequencies observed between YMSM and older males, the correlation plots were adjusted for age to prevent confounding based on this variable.

Samples from 72 of the 77 participants were included in the microbiome analyses as 3 participants had no swab collected, 1 specimen was excluded to avoid potential PCR amplification batch effects, and another specimen was excluded due to small library size of 42 copies. The alpha diversities (measured by the Shannon index) were compared between YMSM and each of the older male cohorts using the Wilcoxon rank-sum test. The correlation between alpha diversity and logAUC p24 concentrations was assessed by Spearman’s rank-order correlation analyses with adjustment for age. Dissimilarity (i.e., distance) between each pair of samples was calculated based on two beta diversity metrics, the Bray-Curtis [based on relative abundance data with a focus on differences of abundant amplicon sequence variants (ASVs)] and Jaccard distances (based on presence-absence data with a focus on differences of less abundant ASVs). These dissimilarities were 1) compared between YMSM and each of the older male cohorts, 2) associated with the top 10 PCs of the correlation matrix for the rectal cellular subset data, and 3) associated with the p24 concentration (as a continuous variable) all using the Permutational Multivariate Analysis of Variance (PERMANOVA) test (32). These results were adjusted for age, and clusters by study groups or dichotomized logAUC p24 were visualized using principal coordinates analysis (PCoA) plots. Following analysis of beta diversities, individual ASVs were analyzed by LDM to detect those with differential abundance or probability of presence across 1) study groups, 2) rectal cellular subset frequencies, or 3) p24 viral replication, respectively (33), while controlling the FDR at the nominal level 10%. Of note, for the analysis of presence-absence data, we used the variant of PERMANOVA and LDM that base on rarefaction to account for variability of the library size but aggregate information over all potential rarefaction replicates without resampling to avoid discarding data (34). For those genera identified as being differentially abundant between study groups, exploratory analyses to assess associations between ASV relative abundance and logAUC p24 concentrations were performed without age adjustment using Spearman’s rank-order correlation, and an adjusted *P* value significance threshold was provided based on the Bonferroni correction method for multiple comparisons. The *Bacteroidaceae/Prevotellaceae* ratios were compared between groups using Kruskal-Wallis test with Dunn’s correction for multiple comparisons. All statistical tests were unadjusted for race, as differences observed that are based on this social construct are more likely a reflection of the physiological effects of systemic racism as opposed to biological differences (35). Data analyses were performed using SAS 9.4 (SAS Institute Inc, NC), R 3.6.0, and GraphPad PRISM 8 (GraphPad Software, CA).

## Results

### The clinical cohort

Thirty-two YMSM aged 19-21 years and 33 AMSM aged 39-50 years engaging in RAI, as well as 12 males aged 40-52 years who had never engaged in RAI (Controls) were enrolled and underwent peripheral blood and rectal biopsy sampling. All study participants were HIV-negative. Demographic and clinical characteristics are presented in **Table 1**. The YMSM reported a lower number of lifetime sexual partners and fewer sexual encounters involving RAI during the prior 12 months compared to AMSM, which supported overall lower lifetime exposure to RAI. There were no differences in the seroprevalence of CMV or HSV-1 between groups. Seropositivity for HSV-2 IgG was significantly higher among the older male cohorts compared to YMSM.

**Table 1:** Demographic and clinical characteristics of HIV-negative YMSM, AMSM, and Control Males.

Characteristic	YMSM (n = 32)	AMSM (n = 33)	Control Males (n = 12)	*P *value
Median age in years (25^th^, 75^th^)	20 (19, 21)	46 (39, 50)	48 (40, 52)	
Race *n* (%) White Black Other	4 (12) 28 (88) 0 (0)	15 (44) 17 (50) 2 (6)	2 (17) 10 (83) 0 (0)	**<0.0001**
Median age at time of sexual debut (25^th^, 75^th^) Total number of sexual partners with whom the participant engaged in RAI during their lifetime, *n* (%) 1 2 to 5 6 to 20 21 to 50 51+ Total number of RAI sexual encounters during the previous 12 months, *n* (%) 0 to 1 2 to 5 6 to 20 21 to 50 51+	18 (16.5, 19) 4 (12.5) 17 (53.1) 7 (21.9) 3 (9.4) 1 (3.1) 10 (31.3) 14 (43.8) 6 (18.8) 0 (0) 2 (6.3)	23 (18, 28) 0 (0) 4 (12.5) 12 (37.5) 11 (34.4) 5 (15.6) 2 (6.1) 6 (18.2) 16 (48.5) 6 (18.2) 3 (9.1)	– – – – – – – – – – –	**<0.0001** **<0.0001** **<0.0001**
Lubricant use, *n* (%)	31 (96.9)	32 (97.0)	–	0.51
Douching before or after RAI, *n* (%)	19 (59.4)	23 (69.7)	–	0.38
CMV IgG positive, *n* (%) HSV-1 IgG positive, *n* (%) HSV-2 IgG positive, *n* (%)	28 (88%) 13 (41%) 3 (9%)	29 (88%) 16 (48%) 17 (52%)	9 (75%) 5 (42%) 7 (58%)	0.39 0.78 **0.0003**

YMSM, young men who have sex with men; AMSM, adult men who have sex with men; RAI, receptive anal intercourse; CMV, cytomegalovirus; HSV, herpes simplex virus. Significant P value < 0.05 for chi square or Fisher’s exact test for proportions. Bold P values are significant at <0.05.

### Rectal mucosa from YMSM supports higher peak HIV p24 production following *ex vivo* challenge compared to AMSM

**Figure 1:**
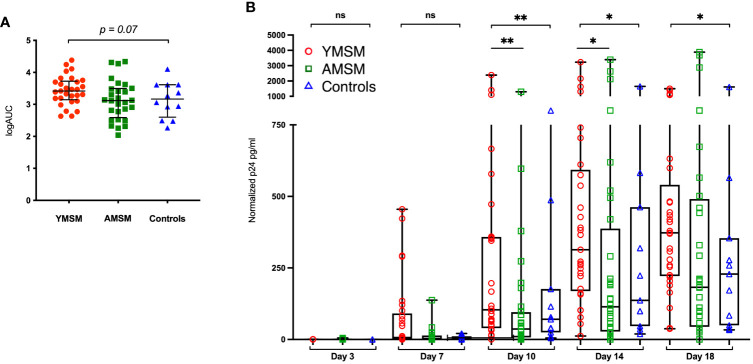
HIV replication in rectal tissues from the *ex vivo* explant challenge experiments. **(A)** There is a trend toward higher median log of the area under the curve (logAUC) p24 values from YMSM compared to the older cohorts. **(B)** Longitudinal normalized p24 concentrations from each cohort for days 3 through 18 post-infection demonstrate higher p24 concentrations among YMSM compared to AMSM for Days 10 and 14 of the challenge. **(A)** Black horizontal lines and vertical ranges represent the median and interquartile range. **(B)** Boxes extend from the 25^th^ to 75^th^ percentiles, whiskers illustrate minimum and maximum values. The horizontal lines within the box plots represent the median. **(A, B)** Statistical analyses were performed using Kruskal-Wallis (KW) One-way ANOVA test (KW *P* values are shown above each plot) followed by pairwise comparisons with Dunn’s test for multiple comparisons (Adjusted *P* values from pairwise comparisons between groups that met significance are designated with asterisks). *P_adj_ ≤ 0.05; **P_adj_ ≤ 0.01; *** *P_adj_* ≤ 0.001. ns, not significant.

The *ex vivo* HIV challenge model provides a unique opportunity to better understand HIV replication dynamics following rectal tissue exposure. Rectal pinch biopsies were obtained from participants *via* rigid sigmoidoscopy and challenged with HIV-1 BaL virus (10^2.8^ TCID_50_). p24 concentrations in the supernatant were assessed over an 18-day period and normalized to biopsy weight. The median logAUC of the normalized p24 concentrations was then calculated for each subject. YMSM had a trend for higher median logAUC p24 values compared to the older male groups (p = 0.07; **Figure 1A**). Notably, these results included early time points (days 3 and 7) where p24 concentrations were generally low or remained beneath the threshold of detection. When comparing normalized p24 concentrations per group by day of assessment, YMSM had significantly increased normalized p24 concentrations compared to AMSM on days 10 and 14 at the peak of viral replication (**Figure 1B**). On day 18, the normalized p24 values were overall significantly different between groups (p=0.048). While YMSM demonstrated the highest median p24 concentration compared to older males on day 18, there were no statistically significant differences in pairwise comparisons between groups adjusted for multiple comparisons. Notably, the 18-day time point may be less relevant given it is post-peak viral replication and occurs at a time when the rectal biopsies have reduced viability. Collectively, these findings suggest that YMSM may have a RM milieu that facilitates higher levels of HIV replication compared to older males.

### Increased CD4+ T cell proliferation and decreased CD4+ and CD8+ pro-inflammatory cytokine producers in rectal tissues from YMSM

**Figure 2:**
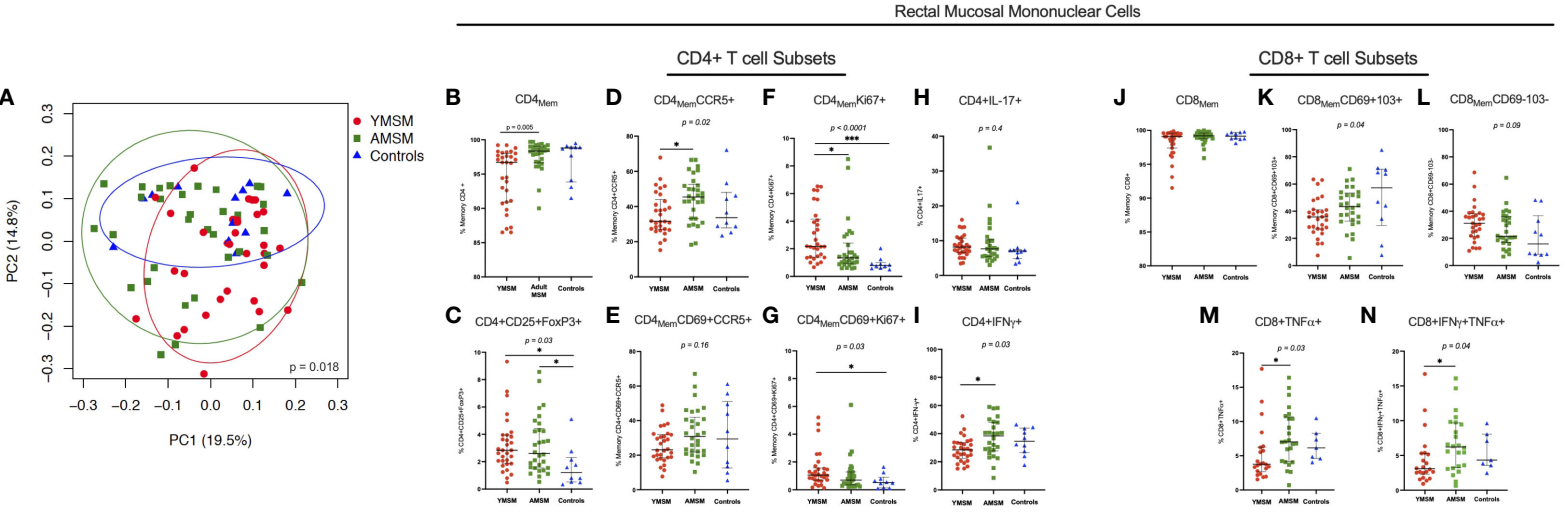
CD4+ and CD8+ T cell composition from the rectal mucosa (RM) demonstrates a distinct cellular immune phenotype among YMSM compared to older males. **(A)** PCA of the rectal CD4+ and CD8+ T cell environment of YMSM, AMSM, and Controls (See **Supplementary Table 1** for comprehensive list of immune cell subsets). *P* value represents global test of difference between the 3 groups from linear decomposition modeling (LDM). Representative dot plots comparing the frequencies of RM immune cell subsets of interest based on linear decomposition model (LDM) findings among YMSM, AMSM, and Controls (all subsets gated on total CD4+ and CD8+ T cells): **(B)** CD4+ memory T cells, **(C)** CD4+CD25+FoxP3+ (T_reg_) cells, **(D)** CD4+CCR5+ memory T cells, **(E)** CD4+CD69+CCR5+ tissue resident memory (T_RM_) cells, **(F)** CD4+Ki67+ memory T cells, **(G)** CD4+CD69+Ki67+ T_RM_ cells, **(H)** CD4+IL-17A+ T cells, **(I)** CD4+IFN-γ+ T cells, **(J)** CD8+ memory T cells, **(K)** CD8+CD69+CD103+ T_RM_ cells, **(L)** CD8+CD69-CD103- non-T_RM_ cells, **(M)** CD8+TNF-α+ T cells, and **(N)** CD8+IFN-γ+TNF-α+ co-expressors. Black horizontal lines and vertical ranges represent the median and interquartile range as reported in **Supplementary Table 2**. Kruskal-Wallis One-Way ANOVA *P* values are shown in italics above the plot; *P* values for pairwise comparisons between groups with Dunn’s adjustment for multiple comparisons are designated with asterisks: *P_adj_ ≤ 0.05; **P_adj_ ≤ 0.01; *** *P_adj_* ≤ 0.001.

Following collagenase digestion of rectal tissues, 26 distinct CD4+ and CD8+ T cell subsets were characterized by flow cytometry based on naïve vs memory phenotype, tissue residence (CD69^+^CD103^+/-^), HIV susceptibility marker expression (CCR5, α4ß7, Ki67) and cytokine production upon mitogen stimulation (IL-17A, IFN-γ, TNF-α) (detailed in **Supplementary Table 1**; representative gating strategies are presented in **Supplementary Figures 1**, **2**). Next, linear decomposition modeling (LDM) (31), a method for testing differential abundance of individual features with FDR control that does not make parametric assumptions about the distribution of the features, was utilized to obtain a global assessment of differences in the rectal T cell compartment between groups. A two-dimensional plot of the principal components analysis (PCA) showed some overlapping among the three cohorts, yet there was distinct clustering of specimens from each study group, suggesting there are distinguishing characteristics of the RM T cell compartment (**Figure 2A**; LDM global *P* value=0.018).

Next, the top 10 cellular subsets identified by LDM (all with adjusted *P* values < 0.20) as contributing to the variability in RM cellular composition between study groups were chosen for further comparative analyses of cellular frequencies between groups with adjustment for multiple comparisons. Despite not reaching the defined p-value cut-off, IL-17A-producing CD4+ T cells were also included in this analysis as current evidence suggests that Th17 cells may play a critical role in early HIV transmission events (36, 37). Also, total memory CD4+ and CD8+ T cell subsets were included to provide context for interpreting differences between groups in the proportional abundance of the phenotypically-characterized CD4+ and CD8+ subpopulations. We found there was significant heterogeneity in the expression of CCR5 (HIV co-receptor) and Ki67 by CD4_Mem_ T cell subsets from younger and older males. YMSM showed lower percentages of rectal CD4+ memory and CD4+CCR5+ memory T cells compared to AMSM (**Figures 2B, D**; **Supplementary Table 2**), and the decreased CCR5 expression was also observed in the comparison of mucosal CD4+CD69+CCR5+ tissue resident memory (T_RM_) cells between groups (**Figure 2E**). In contrast, YMSM demonstrated greater expression of Ki67 by RM CD4_Mem_ T cell subsets compared to the older males (**Figures 2F, G**). In addition, there were higher frequencies of T regulatory (T_reg_; CD4+CD25+FoxP3+) cells among both groups of men engaging in RAI compared to Controls (**Figure 2C**).

In the rectal CD8+ T cell compartment, YMSM had a lower frequency of mucosal CD8+ T_RM_ (CD69+CD103+) cells and a corresponding increased frequency of non-T_RM_ CD8+ T cells compared to older males (**Figures 2K, L** **)** despite similar frequencies of total CD8_Mem_ T cells among the study cohorts (**Figure 2J**).

Among the cytokine producing T cells, YMSM demonstrated significantly reduced frequencies of IFN-γ-producing CD4+ T cells, TNF-α-producing CD8+ T cells, and TNF-α/IFN-γ co-expressing CD8+ T cells compared to AMSM (**Figures 2I, M, N**, respectively). No significant differences in the frequencies of IL-17A-producing CD4+ T cells were observed (**Figure 2H**).

### Distinct RM microbiome composition in YMSM

**Figure 3:**
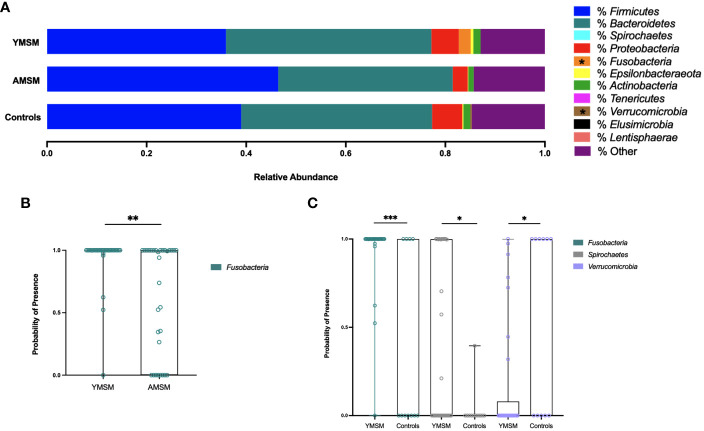
Differences in relative abundance and probability of presence of gut microbiota at phylum level in YMSM compared to the older male cohorts. **(A)** Relative abundance (%) of the 11 most prevalent bacterial phyla. *Fusobacteria* was enriched in YMSM compared to the older male cohorts. YMSM had a lower abundance of *Verrucomicrobia* compared to Control males. Asterisk denotes FDR-adjusted *P* value ≤ 0.10. **(B)** Box and whisker plot showing a higher probability of presence of *Fusobacteria* among YMSM compared to AMSM. **(C)** Box and whisker plot demonstrating higher probability of presence of *Fusobacteria* and *Spirochaetes* and lower probability of presence of *Verrucomicrobia* among YMSM compared to Controls. Dissimilarities in beta diversity between YMSM and older cohorts were detected using the PERMANOVA test, and differential abundance of ASVs were analyzed by the linear decomposition model (LDM). b,c: Boxes extend from the 25^th^ to 75^th^ percentiles, whiskers illustrate minimum and maximum values. *P* values shown are adjusted for multiple comparisons using a false discovery rate of 10%: *P_adj_ ≤ 0.1; **P_adj_ ≤ 0.05; *** *P_adj_* ≤ 0.01.

To examine differences in microbiome composition, mucosal swabs were collected during rigid sigmoidoscopy for 16S rRNA sequencing. After filtering out rare phyla present in fewer than 5 samples, there were 21 phyla out of 30 that remained in the comparative analyses between YMSM and AMSM, and 18 phyla that remained in the comparison between YMSM and Controls. *Fusobacteria* was enriched among samples from YMSM compared to AMSM based on relative abundance (p_adj_ = 0.08; **Figure 3A**) and presence-absence data (p_adj_ = 0.04; **Figure 3B**). Compared to Controls, the microbiome configuration of YMSM was characterized by both a lower abundance (p_adj_ = 0.04; **Figure 3**) and lower probability of presence (p_adj_ = 0.05; **Figure 3C**) of *Verrucomicrobia*. Conversely, the phyla *Fusobacterium* (*P*_adj_ = 0.001) and *Spirochaetes* (*P*_adj_ = 0.099) had a higher probability of presence among YMSM (**Figure 3C**).

As enrichment of *Prevotellaceae* among MSM has been reported previously by our group and others (11–13), we assessed differences in the relative abundance of *Prevotellaceae* and compared the *Bacteroidaceae/Prevotellaceae* (B/P) ratio between cohorts. While there were no differences in relative abundance of *Prevotellaceae* between YMSM and the older male groups, in pairwise comparisons of the B/P ratio, Controls were found to have a significantly higher ratio compared to AMSM (Controls vs AMSM: 15.1 vs 0.33, *P*_adj_ = 0.04) and a higher B/P ratio compared to YMSM that lacked statistical significance (Controls vs YMSM: 15.1 vs 0.36, *P*_adj_ = 0.14).

**Figure 4:**
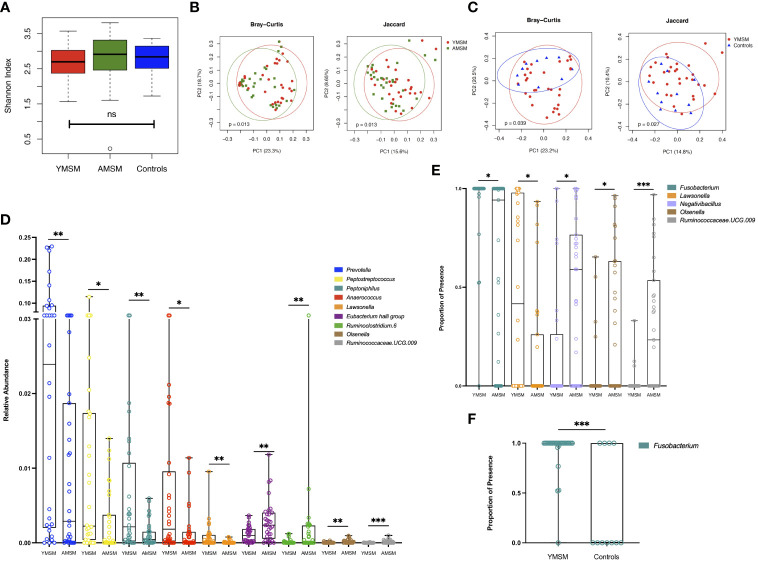
A comparison of bacterial alpha and beta diversity between the YMSM and the older male cohorts. **(A)** Alpha diversity plot, estimated by the Shannon Index, shows no significant differences between groups. Boxes show first and third quartiles, whiskers illustrate values extending from the boxes up to ± 1.5x the interquartile range. **(B)** Principal coordinates analysis (PCoA) projection of Bray-Curtis and Jaccard distance between YMSM and AMSM. **(C)** PCoA projection of Bray-Curtis and Jaccard distance between YMSM and Controls. **(D)** Box and whisker plot showing ASVs that are differentially enriched between YMSM and AMSM based on linear decomposition model (LDM) relative abundance results. **(E)** Box and whisker plot showing differences in ASV enrichment between YMSM and AMSM based on LDM presence-absence data. **(F)** Box and whisker plot showing a higher probability of presence of *Fusobacterium* among YMSM compared to Controls. **(D–F)** Boxes extend from the 25^th^ to 75^th^ percentiles, whiskers illustrate minimum and maximum values. The horizontal lines within the box plots represent the median. *P* values shown are adjusted for multiple comparisons using a false discovery rate of 10%: *P_adj_ ≤ 0.1; **P_adj_ ≤ 0.05; *** *P_adj_* ≤ 0.01.

Next, we focused our attention on the genus level data, in which 424 genera were identified as being present in at least one sample. Taxonomic alpha diversity was estimated using the Shannon Index and demonstrated no significant differences between groups (**Figure 4A**). Dissimilarity in community composition was estimated using the Bray-Curtis and Jaccard indices, and these differences were displayed with Principal Coordinates Analysis (PCoA). For both measures of beta diversity, the microbiome composition of YMSM was significantly dissimilar compared to AMSM (**Figure 4B**) and Controls (**Figure 4C**). LDM analysis was then performed to identify the ASVs contributing to these differences in beta diversity between groups, and the ASVs were filtered to remove the least prevalent taxa, specifically, those present in fewer than 5 samples, to limit the number of pairwise comparisons performed. This resulted in 230 (YMSM vs AMSM) and 193 (YMSM vs AMSM) genera for comparative analyses. Nine ASVs were identified as having differential relative abundance between the MSM cohorts, and five of these genera were more abundant in YMSM compared to AMSM – *Prevotella, Peptostreptococcus, Peptoniphilus, Anaerococcus*, and *Lawsonella* (**Figure 4D**; FDR-adjusted *P* values ≤ 0.10). No differences in taxonomic relative abundance were identified between YMSM and Controls. A comparison of presence-absence data between YMSM and AMSM identified differences involving 5 ASVs with *Fusobacterium* and *Lawsonella* being the two ASVs more likely to be present among YMSM (**Figure 4E**; FDR-adjusted *P* values ≤ 0.10). *Fusobacterium* was also recognized as having a higher probability of presence among YMSM compared to Controls (*P*_adj_ = 0.009; **Figure 4F**).

Additionally, we looked for associations between the microbiome beta diversities and the rectal immune cellular subsets. A global association was found between the top 10 principal components (PCs) of the correlation matrix for the rectal cellular subset data and the Jaccard distance matrix (based on presence-absence statuses) for the microbiome data with adjustment for age (*P* = 0.09). No such association was observed with the microbiome data at the relative abundance scale using the Bray-Curtis distance matrix. Next, we evaluated whether any of the individual cellular subsets of interest, identified by LDM and presented in **Figure 2**, were associated with the microbiome beta diversities. The frequency of T_reg_ positively associated with the relative abundance of *Streptococcus*, a higher probability of presence of *Granulicatella* and *Fusobacterium*, and a lower probability of presence of *Alistipes, Parabacteroides, Bilophila, Tyzzerella*, and *Ruminococcaceae (all P <0.10).*

### Elevated CD4+ T cell proliferation and lower frequencies of pro-inflammatory cytokine producing CD4+ T cells were associated with increased p24 production in the rectal explant challenge model

**Figure 5:**
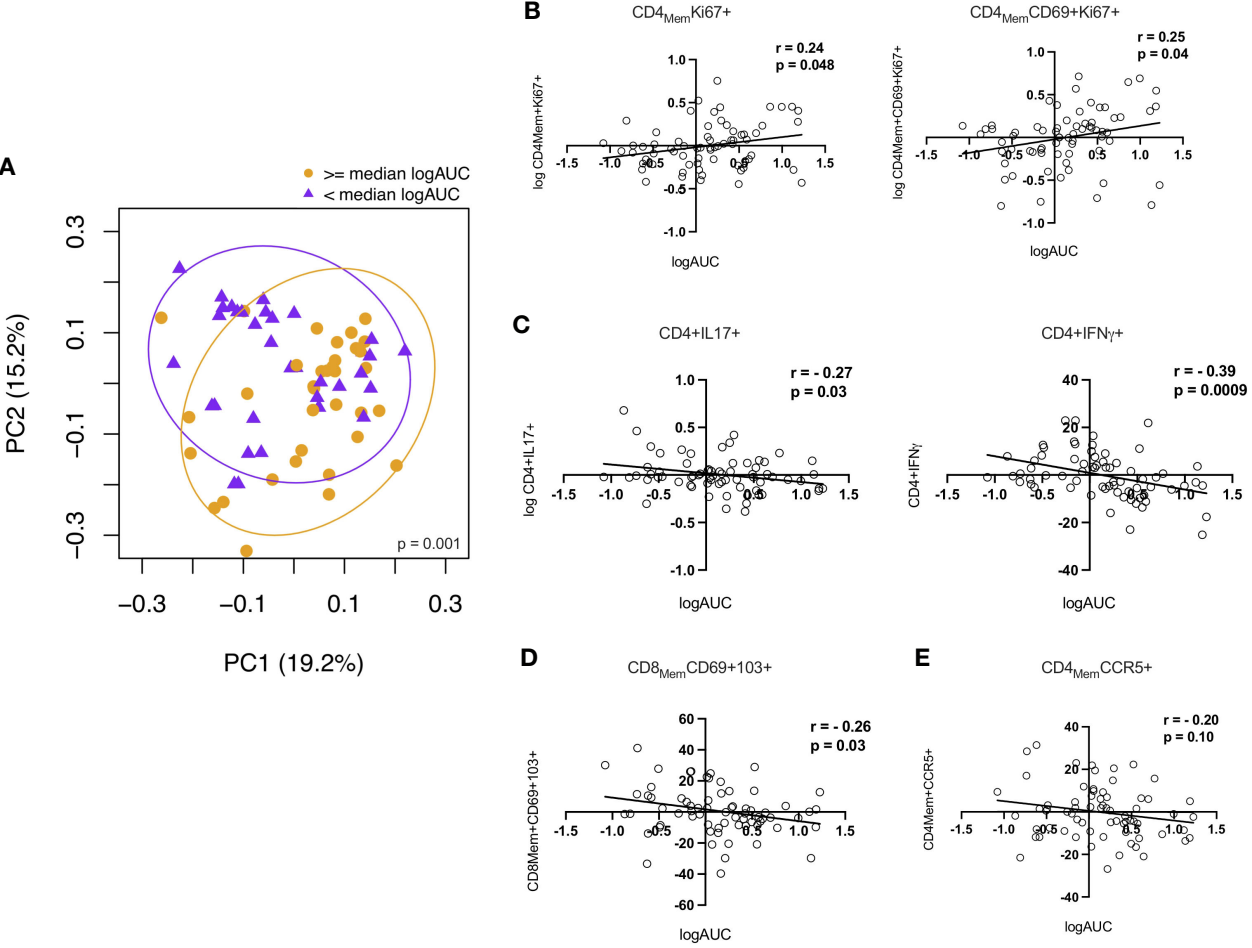
Correlations between CD4+ and CD8+ T cells and p24 viral replication from the explant challenge model. **(A)** Principal components analysis showing a distinct rectal mucosal adaptive immune phenotype depending on high (orange) vs low (purple) HIV viral replication (≥ or < median logAUC of p24 levels). **(B)** Proliferating CD4+Ki67+ T cell subsets demonstrate a positive correlation with viral replication. **(C)** Higher frequencies of IL-17A and IFN-γ-producing CD4+ T cells and **(D)** CD8+ TRM cells were negatively associated with p24 production in the HIV-1 explant challenge model. **(E)** There was no significant association between viral replication and the frequency of CD4+CCR5+ T cells. Spearman rank-order correlation plots were derived from age-adjusted cellular subset and p24 data. Spearman correlation P values significant at < 0.05.

To investigate potential relationships between the immunologic parameters measured in the RM and HIV replication, we first dichotomized logAUC p24 from the *ex vivo* challenge model at the median for all study participants and examined associations with the 26 CD4+ and CD8+ T cell subsets analyzed. PCA showed distinct clustering based on higher vs lower p24 production (**Figure 5A**). Age-adjusted associations between p24 production and the frequencies of the top 10 rectal CD4+ and CD8+ T cell subsets identified by LDM (as described above) showed a positive correlation between p24 production and the Ki67+ total memory and T_RM_ CD4+ T cell subsets (**Figure 5B**). CD8+ T_RM_ cells (**Figure 5D**) and IL-17A- and IFN-γ-producing CD4+ T cells (**Figure 5C**) were negatively correlated with logAUC p24. The proportion of CD4+CCR5+ memory T cells was not significantly associated with p24 production in this model (**Figure 5E**). There were no significant associations between CD8_Mem_ pro-inflammatory cytokine production and *ex vivo* viral replication (data not shown). Although older males were found to have a higher HSV-2 seroprevalence compared to YMSM, there were no statistically significant differences in p24 viral replication or rectal cellular subset frequencies between HSV-2 seropositive and seronegative participants from all groups after adjusting for multiple comparisons.

### HIV replication within the explant challenge model is associated with a distinct microbiome composition

**Figure 6:**
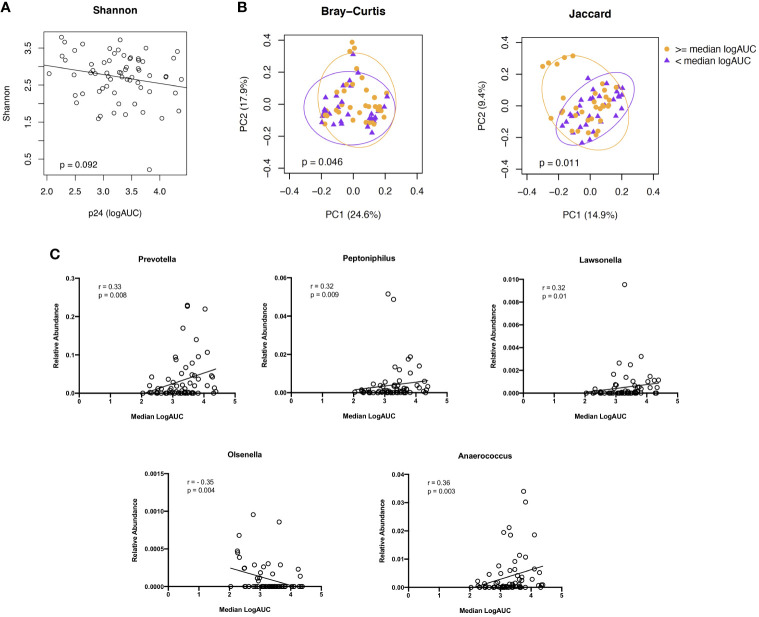
Associations between microbiome composition and *ex vivo* viral replication levels from rectal tissues following HIV challenge. **(A)** Alpha diversity, estimated by the Shannon Index, did not show a significant association with viral replication. **(B)** PCoA plots showing two indices of beta diversity, Bray-Curtis and Jaccard, demonstrated separation based on high (orange) vs low (purple) levels of HIV viral replication (≥ or < median logAUC of p24 concentrations). **(C)** Correlation plots showing significant associations between p24 concentrations and the relative abundance of 5 of the 11 genera identified as having differential abundance between YMSM and AMSM. Raw *P* values shown; when Bonferroni correction for multiple comparisons is applied, adjusted *P* value is significant at <0.005.

As with the cellular subsets, we evaluated associations between microbiome diversity and dissimilarity and higher vs lower levels of viral replication (median logAUC p24 values) from the HIV explant challenge experiments. We found no significant difference in species abundance and evenness, as measured by the Shannon Index, based on the level of viral replication (**Figure 6A**). PCoAs of taxonomic dissimilarity, based on the Bray-Curtis and Jaccard indices, showed distinct clustering for higher vs lower levels of viral replication from HIV-infected rectal tissues (**Figure 6B**). In the corresponding LDM analysis, no specific ASVs were identified as significantly contributing to these differences in beta diversity. Given the concern that adjustment for multiple comparisons for >400 genera may have reduced our power to detect genera of interest moderately associated with HIV viral replication, exploratory analyses were undertaken to assess whether specific taxa of interest (i.e., the 11 genera identified as being dissimilarly abundant and/or present between YMSM and the older male groups in the previous beta diversity analyses) correlated with viral replication. *Prevotella, Peptoniphilus, Lawsonella*, and *Anaerococcus*, which previously had been identified as being enriched among YMSM, were found to be positively associated with viral replication (all *P* ≤ 0.01). *Olsenella*, one of the ASV found to be in higher abundance among AMSM compared to YMSM, demonstrated a negative association with p24 production (P = 0.004; **Figure 6C**). Taken together, these findings provide some evidence to suggest that preferential abundance of certain taxa among YMSM may result in a distinct microbiome composition that influences HIV replication within the rectum.

## Discussion

These data show that the rectal environment of YMSM is characterized by subtle, yet relevant distinctions in the T cell compartment and microbiome composition compared to older males. We found lower levels of CCR5 expression by CD4+ T cells, increased CD4+ T cell proliferation, fewer mucosal CD8+ T_RM_ cells, and decreased frequencies of certain pro-inflammatory cytokine producing CD4+ and CD8+ T cells among YMSM. Furthermore, despite similar levels of species richness, YMSM were observed to have differences in the microbiome composition, compared to the older cohorts, distinguished by enrichment with certain anaerobic taxa, including *Prevotella, Peptoniphilus, Lawsonella, Fusobacterium*, and *Anaerococcus*. Notably, the associations between the rectal T cell immune phenotype and function and microbiome composition of YMSM with higher HIV-1 viral replication in the *ex vivo* explant challenge model suggests these differences could influence HIV replication within RM tissues of these young men.

One potential reason for increased HIV replication among YMSM compared to AMSM in this study is the higher proportion of proliferating CD4_Mem_ T cells in their rectal tissues. HIV preferentially infects activated, proliferating CD4+ cells, as they can accommodate higher levels of viral replication and more efficiently amplify viral spread (38, 39). Our findings, therefore, underscore the importance of CD4+ T cell activation and proliferation in promoting mucosal HIV transmission. As there are no known prior studies specifically characterizing the rectal immune environment of young, HIV-negative men, it is unclear whether a greater proportion of proliferating CD4+ T cells would be seen among adolescents generally or whether this finding could be related to sexual behavior. In our study, AMSM reported a more remote sexual debut, a greater number of RAI partners, and more RAI episodes, and we hypothesize that increased exposure to RAI over time could induce some immune tolerance exemplified by lower levels of inflammation and immune activation. In contrast, sporadic RAI, as reported by the majority of YMSM in our cohort, may promote more robust inflammatory responses during sexual intercourse and lead to increased CD4+ T cell proliferation (40).

HIV co-receptor expression, primarily CCR5, is essential for HIV-1 entry into CD4+ T cells (10, 41). In this study, we observed the highest levels of CCR5 expression on CD4+ T cells from AMSM, and unexpectedly, in our HIV explant challenge model, the percentage of CD4+CCR5+ T cells did not correlate with viral replication. Despite a shared immune phenotype, the CD4+ CCR5+ T cell populations within mucosal tissue sites are diverse and comprised of distinct subsets with specialized functions, including T_RM_, T_reg_, central memory (T_CM_), effector memory (T_EM_), and terminally differentiated T cells that re-express CD45RA (T_EMRA_) (42). Of note, differential CCR5 expression among naïve and memory CD4+ subsets from HIV-uninfected individuals has been observed, yet the levels of CCR5 expression did not predictably correlate with HIV infectivity (43). Likewise, despite the expression of CCR5, T_reg_ cells are not preferentially targeted by HIV (44), likely in part due to FoxP3-mediated inhibition of HIV-1 transcription (45, 46). Therefore, we hypothesize that CCR5 expression, while essential for HIV-1 infection of CD4+ T cells by CCR5-using virus, must be considered in conjunction with other factors, including the cellular subtype and the activation status of a cell, when assessing overall HIV susceptibility. Our data show that while a higher percentage of rectal CD4+ T cells from AMSM expressed CCR5, the proliferation status of these cells, which was higher among YMSM, was a more relevant predictor of HIV replication in the explant challenge model.

CD4+ T cells that secrete IL-17 (Th17 cells) have been proposed by our group and others as initial targets for HIV during rectal transmission (37). We previously characterized higher frequencies of Th17 cells and pro-inflammatory CD8+ T cells within the rectal tissues of men engaging in condomless RAI compared to control males who had never engaged in RAI and hypothesized that these differences in cellular composition may be contributing to rectal HIV transmission (11). In this study, however, we found no significant differences between groups in the frequencies of rectal CD4+IL-17+ T cells, and higher frequencies of IL-17A and IFN-γ-producing CD4+ T cells were negatively associated with HIV-1 viral replication in the explant challenge model. In contrast to the prior work, statistical power in this study was limited to detect a difference between MSM and Controls as this was not the primary comparison of interest, and we did not perform rectal sampling based on timing of condomless RAI exposures as we did in the prior study. Importantly, the prior work did not involve *ex vivo* HIV explant challenge experiments; therefore, this study is unique in its assessment of associations between the rectal cellular composition and viral replication following *ex vivo* infection of rectal tissues. It is possible that the frequency of Th17 cells is not a critical factor in promoting early viral propagation, as our data supports cellular proliferation as being a major determinant of HIV replication within rectal tissues. Considering the myriad of factors influencing HIV replication within the complex rectal tissue environment, we hypothesize that the modest associations between immune cell subsets and viral replication are relevant, and we posit that these CD4+ T cells with a Th1- and Th17-like phenotype may be secreting other cytokines or chemokines with the ability to inhibit HIV viral replication, such as RANTES, macrophage inflammatory protein (MIP)-1α, and MIP-1β. These β chemokines bind to CCR5, competitively block viral interaction with the HIV co-receptor, and may promote the down-regulation of CCR5 from the host-cell surface (47). As multiple cytokines/chemokines are secreted in response to HIV transmission (48), it is likely that some of these factors have the capacity to limit viral propagation at mucosal sites. Ongoing investigation into the specific secretory functions of adaptive immune subsets in the RM could lead to the identification of HIV suppressive factors and potential targets in the development of future HIV prevention interventions.

The extent to which the gut microbiome directly or indirectly influences HIV transmission and pathogenesis remains an important, yet unanswered, question in the literature. There is evidence that the microbiome plays a critical role in regulating the gut immune environment by impacting the cellular composition and levels of immune activation (49–51). Here, we observed an association between the proportion of T_reg_ cells within the rectal tissues and the microbiome composition, a relationship previously described in the literature (52, 53). Consistent with prior studies (12, 13), the stool microbiota of our MSM cohorts demonstrated a lower ratio of *Bacteroidaceae* to *Prevotella*ceae compared to Controls. This has relevance for rectal HIV transmission as enrichment of *Prevotellaceae* has been associated with levels of immune activation, increased frequencies of HIV target cells, and enrichment for gene expression signatures associated with mucosal injury and repair (11, 50). Here, we have demonstrated that the microbiome composition of YMSM is characterized by enrichment of specific anaerobic genera (i.e., *Prevotella, Peptoniphilus, Lawsonella*, and *Anaerococccus*), and exploratory analyses showed that higher relative abundance of these ASVs were positively associated with HIV viral replication in the explant model. While caution should be employed when comparing microbiome characteristics between different mucosal sites, penile bacterial communities enriched with *Prevotella, Peptostreptococcus* and *Peptoniphilus* were shown to be associated with increased HIV acquisition risk mediated through high tissue density of HIV target cells and elevated tissue levels of IL-8 and α-defensin (54). While our data do not directly link the gut microbiome composition to RM immune cell subsets found to be associated with viral replication in our model, we hypothesize that the microbiome composition observed among YMSM could be influencing other aspects of the rectal immune environment, such as innate immunity, the functionality of adaptive immune cells, and/or production of inflammatory markers. Therefore, the microbiome may be impacting rectal HIV susceptibility through other immunologic mechanisms not examined here.

This study had some limitations. As we did not include a control group of young men not engaging in RAI, we were unable to determine the unique mechanisms of age or sexual behavior characteristics on the RM immune environment of YMSM. However, we argue that exact mechanisms (age vs sexual behavior) may be less relevant given both factors are simultaneously operating in YMSM to potentially increase their risk for HIV acquisition. It is possible that the AMSM in this study, who have remained HIV-negative for an extended period of time compared to the YMSM, may benefit from unspecified host protective factors against HIV infection (i.e., survival bias). Viral suppressive factors that may be secreted by CD4+ and CD8+ T cells were not exhaustively evaluated here but will be important in further analyses to better understand the full arsenal of defenses against rectal HIV transmission. Our study sample size was modest and lacked statistical power to assess all possible immunologic associations. Despite these challenges, we posit that the associations with large effects described here are those most likely to have clinical implications for human HIV transmission.

In summary, we have identified distinct features of the rectal immunologic and microbial milieu of YMSM that could play a role in facilitating rectal HIV transmission. To our knowledge, this is the first study of its kind to comprehensively evaluate the RM immune environment and microbiome composition of HIV-negative at-risk YMSM and to compare those results directly with older male cohorts. These findings will serve as a basis for future mechanistic studies that further elucidate rectal HIV susceptibility among YMSM as this could inform the optimization of biomedical HIV prevention modalities that provide a direct, protective benefit for this population.

## Data availability statement

The raw data supporting the conclusions of this article will be made available by the authors, without undue reservation. The 16S data presented in the study are deposited in the NCBI Sequence Read Archive (SRA), accession number PRJNA881329.

## Ethics statement

The studies involving human participants were reviewed and approved by Emory University Institutional Review Board. The patients/participants provided their written informed consent to participate in this study.

## Author contributions

CFK is responsible for conception of the work, funding acquisition, oversight and conduct of the human subjects protocol, oversight of the laboratory and data analyses and interpretation, and writing/editing of the manuscript. CGA performed data analyses and wrote the manuscript. PA assisted with study design, performed laboratory assays and provided critical review of the manuscript. SS performed laboratory assays, contributed to data analyses, and provided critical review of the manuscript. PM performed laboratory assays and provided critical review of the manuscript. RAA contributed to data analyses and provided critical review of the manuscript. Y-JH and ZZ provided statistical expertise, contributed to data analyses, and provided critical review of the manuscript. AC contributed to data analyses and provided critical review of the manuscript. RRA assisted with data analyses and provided critical review of the manuscript. All authors contributed to the article and approved the submitted version.

## Funding

The project described was supported by the following funding sources: R01 AI128799-01 (CFK), T32 DK108735 (CGA), K12 HD085850 (CGA), and The Emory Center for AIDS Research P30 AI050409 (CFK, CGA).

## Acknowledgments

We thank the study volunteers for their participation in this research.

## Conflict of interest

CFK has received research grants from Gilead Sciences, ViiV, Moderna, Novavax, and Humanigen. PA is/was employed by Pfizer, Inc.

The remaining authors declare that the research was conducted in the absence of any commercial or financial relationships that could be construed as a potential conflict of interest.

## Publisher’s note

All claims expressed in this article are solely those of the authors and do not necessarily represent those of their affiliated organizations, or those of the publisher, the editors and the reviewers. Any product that may be evaluated in this article, or claim that may be made by its manufacturer, is not guaranteed or endorsed by the publisher.
