# Supplementary material for: The rectal mucosal immune environment and HIV susceptibility among young men who have sex with men
Source: Front Immunol. 2022 Oct 20;13:972170. doi: 10.3389/fimmu.2022.972170 (PMC9631201; doi:10.3389/fimmu.2022.972170)
Supplement: Supplementary file 1 [file DataSheet_1.docx]

Supplementary Material

**Mean Substitution for Missing Cellular Subset Data**

To identify rectal cellular subsets for this study, two flow cytometry panels were utilized – one that distinguished tissue residence and HIV susceptibility marker expression of CD4+ and CD8+ T cells and a second for identifying cytokine producers. If the yield from a rectal biopsy specimen was <1.5 million cells, then only the first panel was performed, which resulted in some missing values in the frequencies of CD4+ and CD8+ cytokine producing subsets. Also, during flow cytometric data analysis, cellular subpopulation data was excluded if the total CD4+ or CD8+ events from that specimen yielded <800 events. A cutoff of <500 total CD4+ or CD8+ T cell events was applied for cytokine-producing subpopulations. Missing data were imputed by the mean of the observed data in the same cellular subset. Using the < 800 event threshold, one participant’s CD4+ subpopulation data were excluded and the CD8+ subpopulation data from 4 participants were excluded. Using the <500 event threshold for cytokine-producing subsets, the CD4+ data from 3 participants and the CD8+ data from 14 participants were excluded. The minimum and maximum proportions of all missing data for cellular subsets were 6.5% and 31.2%, respectively, and the overall median proportion of missingness among all 26 subsets was 11.1%.

**Supplementary Table 1. 26 distinct rectal mucosal CD4+ and CD8+ T cells subsets identified based on immune phenotype and function**

| Total CD3+ | Total CD8+ |
| --- | --- |
| Total CD4+ | Memory CD8+ |
| Memory CD4+ | Ki67+ CD8_Mem_ |
| α4β7+ CD4_Mem_ | CD69+ CD103+ CD8_Mem_ |
| CCR5+ CD4_Mem_ | Ki67+ CD69+ CD103+ CD8_Mem_ |
| Ki67+ CD4_Mem_ | CD69- CD103- CD8_Mem_ |
| CD69+ CD4_Mem_ | IL17A+ CD8+ |
| α4β7+ CD69+ CD4_Mem_ | IFN-γ+ CD8+ |
| CCR5+ CD69+ CD4_Mem_ | TNF-α+ CD8+ |
| Ki67+ CD69+ CD4_Mem_ | IFN-γ+/ TNF-α+ CD8+ co-expressors |
| CD25+ FoxP3+ CD4+ (Treg) |  |
| IL17A+ CD4+ |  |
| IL22+ CD4+ |  |
| IFN-γ+ CD4+ |  |
| TNF-α+ CD4+ |  |
| Th1 Th17 CD4_Mem_ |  |

Abbreviations: IFN-γ, interferon gamma, TNF, tumor necrosis factor

**Supplementary Table 2. The distribution of pre-defined rectal mucosal CD4+ and CD8+ T cell subsets identified by LDM among YMSM, AMSM, and Control males**

| **Immunological index** | ***n*** | | **Median**  **(25^th^, 75^th^)** | **Kruskal-Wallis Test *P*-value** | ***P_adj_* for Pairwise Comparisons (vs YMSM*)*** |
| --- | --- | --- | --- | --- | --- |
| *Rectal CD4^+^ cells* | | | | | |
| % CD4_Mem_  YMSM  AMSM  Control males | 31  30  10 | 96.7 (90.9, 98.0)  98.4 (96.7, 99.0)  98.8 (93.9, 99.0) | | **0.003** | --  **0.005**  0.06 |
| % CCR5^+^CD4_Mem_  YMSM  AMSM  Control males | 30  30  10 | 31.7 (27.1, 44.0)  45.5 (33.2, 52.6)  33.7 (27.9, 48.0) | | **0.02** | --  **0.02**  >0.99 |
| % Ki67^+^CD4_Mem_  YMSM  AMSM  Control males | 30  30  10 | 2.2 (1.4, 4.2)  1.3 (0.9, 2.4)  0.8 (0.6, 1.0) | | **<0.0001** | --  **0.02**  **<0.0001** |
| % CCR5^+^CD69*^+^*CD4_Mem_  YMSM  AMSM  Control males | 30  30  10 | 23.1 (18.4, 31.9)  30.8 (21.9, 42.0)  29.4 (12.6, 51.1) | | 0.16 | --  0.17  >0.99 |
| % Ki67^+^CD69*^+^*CD4_Mem_  YMSM  AMSM  Control males | 30  30  10 | 1.1 (0.7, 1.6)  0.7 (0.4, 1.3)  0.5 (0.1, 0.9) | | **0.03** | --  0.25  **0.04** |
| % CD25^+^ FoxP3^+^  YMSM  AMSM  Control males | 31  30  10 | 2.8 (1.9, 4.0)  2.6 (1.5, 4.4)  1.2 (0.5, 2.3) | | **0.03** | --  >0.99  **0.03** |
| *Rectal CD8^+^ cells* | | | | | |
| % CD8_Mem_  YMSM  AMSM  Control males | 31  30  10 | 99.1 (97.4, 99.6)  99.2 (98.8, 99.6)  99.2 (98.8, 99.7) | | 0.25 | --  0.38  0.74 |
| % CD69^+^103^+^CD8_Mem_  YMSM  AMSM  Control males | 29  28  10 | 35.8 (26.9, 41.1)  43.4 (32.7, 53.4)  57.2 (29.5, 70.8) | | **0.04** | --  0.17  0.06 |
| % CD69^-^CD103^-^CD8_Mem_  YMSM  AMSM  Control males | 29  28  10 | 31.1 (21.6, 38.1)  21.4 (17.0, 36.0)  16.0 (8.3, 36.6) | | 0.09 | --  0.41  0.13 |
| *Stimulated rectal CD4^+^ cells* | | | | | |
| % IL-17A^+^  YMSM  AMSM  Control males | 30  26  10 | 8.2 (6.6, 10.7)  7.6 (5.5, 10.3)  7.0 (4.8, 7.5) | | 0.35 | --  >0.99  0.45 |
| % IFN-γ^+^  YMSM  AMSM  Control males | 30  26  10 | 28.5 (22.5, 33.6)  38.4 (27.8, 48.1)  34.7 (26.6, 43.9) | | **0.03** | --  **0.03**  0.30 |
| *Stimulated rectal CD8^+^ cells* | | | | | |
| % TNF-α^+^  YMSM  AMSM  Control males | 22  24  7 | 3.8 (2.7, 6.3)  7.0 (4.1, 10.7)  6.1 (4.6, 8.3) | | **0.03** | --  **0.03**  0.37 |
| % IFN-γ^+^/TNF-α^+^  YMSM  AMSM  Control males | 22  24  7 | 3.1 (2.3, 5.3)  6.2 (3.2, 9.7)  4.3 (3.6, 8.0) | | **0.04** | --  **0.04**  0.53 |

Abbreviations: YMSM, young men who have sex with men, AMSM, adult men who have sex with men. IFN-γ, interferon gamma, TNF, tumor necrosis factor. Bold items are significant *P* values (<0.05).

**
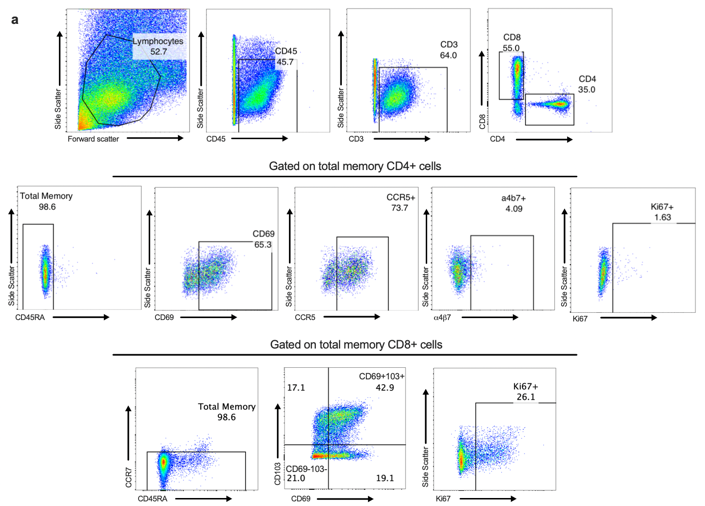
**

**Supplementary Figure 1. Representative gating strategy for rectal mucosal mononuclear cells.** Lymphocytes were identified by forward and side scatter. CD45+ cells were then isolated, followed by CD3+ cells which were separated into CD4+ and CD8+ subsets. Memory CD4 cells were identified by excluding CD45RA+ cells. CD69 marker was then used to divide CD4+ cells into tissue resident and non-tissue resident populations. Memory CD8 + cells were designated as being CCR7^-^ and CD45RA^+/-^. Among memory CD8+ T cells, both CD69 and CD103 markers were used to designate these populations. Memory CD4+ populations, including tissue resident and non-tissue resident subsets were then assessed for expression of CCR5, α4β7, and Ki67.


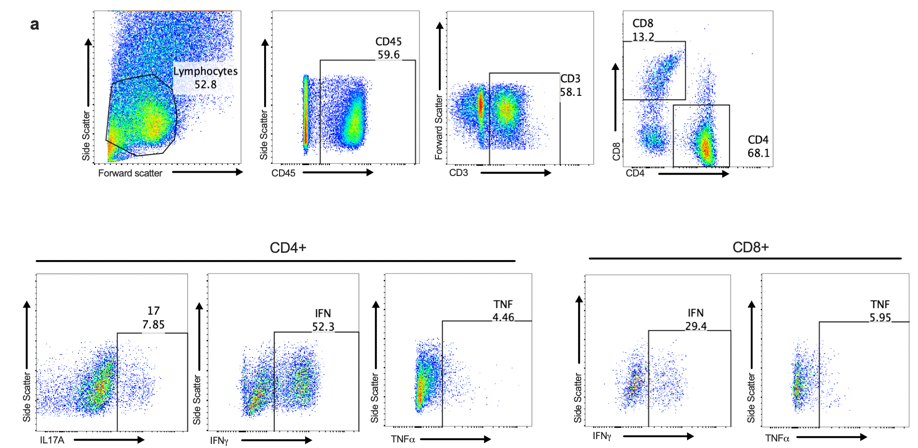


**Supplementary Figure 2. Representative gating strategy to detect cytokine-positive rectal mucosal CD4+ and CD8+ T cells.** Rectal CD4+ and CD8+ MMCs were stimulated for 4 hours with PMA/Ionomycin and stained for indicated cytokines. Live cells were identified by live/dead staining and lymphocytes were identified by forward and side scatter. CD45+ cells were then isolated, followed by CD3+ cells which were separated into CD4+ and CD8+ subsets. Stimulated CD4+ T cells were assessed for IL-17A, IFN-γ, and TNF-α cytokine production and stimulated CD8+ T cells for IFN-γ and TNF-α. Abbreviations: MMC, mucosal mononuclear cells, PMA, phorbol myristate acetate, IFN-γ, interferon gamma, TNF, tumor necrosis factor.
